# The microbiome bone axis in osteomyelitis: integrating evidence from bone oral and gut microbiota

**DOI:** 10.20517/mrr.2026.63

**Published:** 2026-09-29

**Authors:** Chanon Kunasol, Chaloemwut Piyaphattachai, Jirawat Saengsin, Nipon Chattipakorn, Siriporn C. Chattipakorn

**Affiliations:** ^1^Neurophysiology Unit, Cardiac Electrophysiology Research and Training Center, Faculty of Medicine, Chiang Mai University, Chiang Mai 50200, Thailand.; ^2^Center of Excellence in Cardiac Electrophysiology Research, Chiang Mai University, Chiang Mai 50200, Thailand.; ^3^Pediatric Unit, Department of Orthopaedics, Faculty of Medicine, Chiang Mai University, Chiang Mai 50200, Thailand.; ^4^Cardiac Electrophysiology Unit, Department of Physiology, Faculty of Medicine, Chiang Mai University, Chiang Mai 50200, Thailand.; ^5^The Academy of Science, The Royal Society of Thailand, Bangkok 10210, Thailand.; ^6^Department of Oral Biology and Diagnostic Sciences, Faculty of Dentistry, Chiang Mai University, Chiang Mai, 50200 Thailand.

**Keywords:** Osteomyelitis, dysbiosis, microbiome-bone axis, chronic bacterial osteomyelitis, chronic nonbacterial osteomyelitis

## Abstract

Osteomyelitis is an inflammatory bone disorder that includes both infectious and noninfectious forms and remains a major cause of bone destruction, impaired skeletal repair, and long-term disability. Although bacterial infection is the predominant cause, disease progression is influenced by interactions among microorganisms, host immunity, inflammation, and bone remodeling. This review critically evaluates current evidence from genome-wide association and Mendelian randomization studies, clinical investigations, and experimental animal models. Clinical studies demonstrate that osteomyelitic bone contains polymicrobial communities enriched with anaerobic and biofilm-forming bacteria, whereas oral microbial dysbiosis differs between chronic bacterial and chronic nonbacterial osteomyelitis. Experimental studies indicate that gut microbial dysbiosis is associated with impaired intestinal barrier function, altered microbial metabolites, systemic inflammation, and delayed bone repair. Current evidence therefore suggests that microbial communities detected at local sites are associated with infection, biofilm formation, and local inflammatory responses, whereas the gut microbiome may influence systemic immunity, inflammatory tone, and bone remodeling. Although microbiome-targeted interventions have shown beneficial effects in experimental models, current evidence remains largely associative, and mechanistic and longitudinal studies are limited. A better understanding of microbiome-host interactions may facilitate the development of microbiome-based strategies for the prevention and treatment of osteomyelitis.

## INTRODUCTION

Osteomyelitis (OM) is an inflammatory bone disorder that can arise from both infectious and non-infectious causes, with bacterial infection being the most common etiology. This disease is associated with substantial morbidity and remains a major clinical challenge despite advances in antimicrobial therapy and surgical management. Osteomyelitis can occur in both acute and chronic forms. Chronic osteomyelitis is characterized by persistent inflammation, progressive bone destruction, impaired bone healing, and recurrent infection, resulting in chronic pain, skeletal deformity, and functional impairment^[1,2]^. The incidence rate of osteomyelitis increased from 11.4 to 24.4 cases per 100,000 person-years between 1969-1979 and 2000-2009^[3]^. This increase has been attributed to the aging of the population, the increasing prevalence of diabetes mellitus, and the emergence of antimicrobial resistance. Infectious osteomyelitis most commonly develops through hematogenous spread but can also occur following trauma, fractures, or orthopedic surgery. *Staphylococcus aureus* remains the predominant causative pathogen^[4,5]^. Its ability to form biofilms on bone surfaces protects the bacteria from host immune defenses and limits antibiotic penetration, contributing to persistent infection and treatment failure.

Osteomyelitis can be broadly classified into infectious and noninfectious forms^[6]^. Infectious osteomyelitis is caused by microbial invasion of bone tissue, whereas chronic nonbacterial osteomyelitis (CNO) is a rare autoinflammatory bone disorder characterized by sterile bone inflammation. Chronic recurrent multifocal osteomyelitis (CRMO) represents a recurrent multifocal form within the CNO spectrum^[6]^. Unlike infectious osteomyelitis, CNO develops in the absence of detectable pathogens and is thought to result from dysregulation of innate immune responses. Current treatment of CNO is primarily directed toward the control of inflammation and the prevention of long-term skeletal complications^[7]^. Although infectious osteomyelitis and CNO differ in etiology, both disorders are characterized by persistent inflammation, bone destruction, and impaired bone remodeling^[4,7]^. Recent evidence suggests that the microbiome may be associated with these pathological processes and may influence host immunity, inflammatory responses, and bone homeostasis^[8-11]^.

The human microbiome plays an essential role in the maintenance of host health through the regulation of immune function, inflammatory responses, and tissue homeostasis. In recent years, increasing attention has been directed toward the microbiome-bone axis, which describes the interaction between microbial communities and bone physiology^[1,11-13]^. Microbiota-derived metabolites, including short-chain fatty acids (SCFAs), together with immune signaling pathways, have been shown to regulate osteoblast differentiation, osteoclast activity, and bone remodeling^[14,15]^. Dysbiosis of the microbiome may therefore be associated with chronic inflammation, impaired bone repair, and skeletal disorders. However, whether these microbial alterations contribute directly to osteomyelitis progression or occur secondary to infection and inflammation remains unclear.

Emerging evidence suggests that alterations in the skin, oral, bone, and gut microbiome are associated with susceptibility to osteomyelitis, disease progression, and treatment response^[9,10,16]^. However, the evidence varies considerably across body sites, disease subtypes, and study designs. Studies ranging from genome-wide association studies (GWAS) and Mendelian randomization analyses to clinical investigations and experimental animal models have identified distinct microbial signatures associated with osteomyelitis^[17-20]^. In addition, microbiome-targeted interventions, including probiotics, prebiotics, dietary modulation, and microbial metabolites, have shown therapeutic potential in experimental models^[21-25]^. However, these findings remain fragmented because most studies have focused on individual body sites or specific disease subtypes. Consequently, the interactions among different microbiome niches during osteomyelitis remain poorly understood.

Therefore, this review critically evaluates current evidence linking the skin, oral, bone, and gut microbiomes to osteomyelitis from genetic prediction studies, clinical investigations, and experimental models. We discuss potential mechanisms linking microbial alteration to inflammation, immune regulation, biofilm formation, and bone remodeling, and use the findings to propose an integrated microbiome-bone axis framework for osteomyelitis. The potential of microbiome-targeted interventions as adjunctive approaches in osteomyelitis is also discussed.

The literature was searched in the PubMed database from inception to 31 May 2026 using the search terms “**Microbiota**” AND “**Osteomyelitis**”. The search identified 68 publications, which were screened for relevance to the microbiota and osteomyelitis. Of these, 29 publications were considered relevant, comprising 21 original research articles and eight review articles. The original research articles included five GWAS studies, ten clinical studies, and six animal studies. The literature search and study selection process is summarized in Supplementary Figure 1. This review was conducted as a narrative review rather than a systematic review.

## OSTEOMYELITIS-ASSOCIATED MICROORGANISMS: EVIDENCE FROM DATABASE PREDICTION

Genome-wide association studies (GWAS) and Mendelian randomization (MR) have been applied to identify microbial taxa that may be associated with susceptibility to osteomyelitis [Table 1]. Previous studies have suggested that genetically predicted variation in the microbiota of both the skin and gut may be associated with the risk of osteomyelitis based on genetic association analyses^[17-20]^. However, most of the available evidence is derived from the United Kingdom Biobank. Therefore, these findings may not be generalizable to other populations, particularly Asian and Southeast Asian populations. Differences in ancestry, age, and metabolic conditions, including diabetes, may also influence microbiota composition and the observed associations with osteomyelitis. Further studies in different populations and clinical settings are needed to confirm these findings.

**Table 1:** Microbiome-associated genetic risk of osteomyelitis: GWAS and mendelian randomization studies

**Model (N)**	**Microbiota alteration**	**Interpretation**	**Ref**
**Skin microbiome**			
Osteomyelitis (4,836) *vs.* Controls (481,648)	**↑ Osteomyelitis risk** *Staphylococcus*, *Propionibacterium*, *Corynebacterium*, *Micrococcaceae* **↓ Osteomyelitis risk** *Clostridiales*, *C. glutamicum*, *Finegoldia*, *Betaproteobacteria*	Skin microbiome-associated taxa were genetically linked to osteomyelitis susceptibility	[17]
**Gut Microbiome**			
Osteomyelitis (4,836) *vs.* Controls (481,648)	**↑ Osteomyelitis risk** Bacilli, Bacteroidia, *Butyricimonas*, Lactobacillales, Bacteroidales **↓ Osteomyelitis risk** *Lachnospira*,Bacteroidales S24.7	Gut microbiota-associated taxa were associated with osteomyelitis susceptibility in MR analysis	[18]
Osteomyelitis (4,836) *vs.* Controls (481,648)	**↑ Osteomyelitis risk** **Bacilli**	Class *Bacilli* was associated with increased osteomyelitis susceptibility in MR analysis	[19]
Osteomyelitis (4,836) *vs.* Controls (481,648)	**↑ Osteomyelitis risk** *Butyricimonas*, *Coprococcus* 3, *Tyzzerella* 3 **↓ Osteomyelitis risk** *Lachnospira*	Gut microbiota-associated taxa were associated with osteomyelitis susceptibility in MR analysis	[20]

All Mendelian randomization analyses used osteomyelitis GWAS summary statistics from the UK Biobank dataset. ↑: increased; ↓: decreased. GWAS: Genome-wide association study; MR: Mendelian randomization.

With regard to the skin microbiome, genetically predicted *Staphylococcus*, *Propionibacterium*, *Corynebacterium*, and members of the Micrococcaceae family were associated with an increased risk of osteomyelitis^[17]^. In contrast, Clostridiales, *Corynebacterium glutamicum*, *Finegoldia*, and *Betaproteobacteria* were associated with a reduced risk^[17]^. The association with *Staphylococcus* is biologically plausible because *Staphylococcus aureus* invades osteoblasts and stimulates the production of pro-inflammatory cytokines, leading to enhanced RANKL-mediated osteoclastogenesis, progressive bone destruction, and persistent osteomyelitis^[26,27]^. In addition, its ability to form biofilms and survive intracellularly promotes immune evasion and antibiotic resistance, further contributing to chronic infection^[26]^. However, these findings are based on genetic prediction and have not been directly validated by skin microbiome profiling in patients with osteomyelitis. Therefore, further clinical and experimental studies are required to confirm these associations.

Several GWAS-based MR studies have also identified associations between the gut microbiota and the risk of osteomyelitis^[18-20]^. The taxa belonging to the class Bacilli were consistently associated with increased susceptibility to osteomyelitis across independent analyses^[18,19]^. Additional taxa associated with an increased risk included *Bacteroidia*, *Butyricimonas*, Bacteroidales, and Lactobacillales^[18]^, as well as *Coprococcus* 3 and *Tyzzerella* 3^[20]^. In contrast, Bacteroidales S24.7 and *Lachnospira* were associated with a reduced risk of osteomyelitis^[18]^, with the protective association of *Lachnospira* also reported in an independent analysis^[20]^. The effect sizes, 95% CIs, and P-values for these associations are summarized in Supplementary Table 1. The protective association of *Lachnospira* is biologically plausible because this genus is a major producer of SCFAs, in particular butyrate, which helps in the maintenance of intestinal barrier integrity, the regulation of immune homeostasis, suppression of pro-inflammatory cytokine production, and inhibition of osteoclastogenesis. Collectively, these genetic associations suggest a potential relationship between gut microbial composition and osteomyelitis susceptibility; however, these associations should not be interpreted as definitive evidence of causality. Although MR can reduce confounding and reverse causation, its interpretation depends on the validity of the genetic instruments. Horizontal pleiotropy may also affect the observed associations if genetic variants influence osteomyelitis through pathways other than the microbial taxa of interest. Therefore, these findings should be interpreted with caution.

The database-driven prediction studies provide preliminary evidence that both skin and gut microbiota may be associated with susceptibility to osteomyelitis. However, these analyses identify microbial taxa associated with genetic predisposition rather than providing direct evidence from patients with osteomyelitis. Therefore, validation through clinical microbiome studies, experimental animal models, and mechanistic investigations is still required to confirm the biological relevance of these predicted associations. Future studies integrating genetic prediction with longitudinal microbiome profiling and functional analyses are needed to further investigate the potential causal relationships between the microbiome and osteomyelitis.

## BONE MICROBIOME AND OSTEOMYELITIS: CLINICAL EVIDENCE

Clinical studies have demonstrated that osteomyelitis is associated with substantial alterations in the bone microbiome, as summarized in Table 2. Rather than being caused by a single pathogen, many osteomyelitic bone lesions contain complex microbial communities. Previous clinical studies investigating the bone microbiota in osteomyelitis have analyzed samples obtained from the affected skeletal sites, including foot bones in diabetic foot osteomyelitis (DFO) and jawbone tissues in chronic osteomyelitis of the jaw (COMJ)^[9,11,28-30]^. Dı´az-Velis *et al.* reported that microbes from bone samples from patients with DFO were predominantly composed of members of the Proteobacteria, Bacteroidetes, Firmicutes, and Actinobacteria phyla, supporting the polymicrobial nature of the disease^[9]^. In addition, spatial heterogeneity of the bone microbiome was observed within chronic DFO lesions, with distinct microbial communities detected across different bone regions, suggesting that the local microenvironment may influence microbial distribution and persistence^[9]^. These findings are consistent with those of van Asten *et al.*, who reported that osteomyelitic bone samples frequently contain polymicrobial communities and that the presence of multiple bacterial taxa was associated with an increased likelihood of bone infection^[11]^.

**Table 2:** Bone microbiomes in osteomyelitis: clinical studies

**Model (N)**	**Bone site**	**Bone microbiota alteration**	**Other parameters**	**Interpretation**	**Ref**
DFO (7)	Foot bone	**Dominant Phyla:** Proteobacteria, Bacteroidetes, Firmicutes, Actinobacteria	**Acute OM:** ↑ PMNs, **Chronic OM:** ↑ Lymphocytes/plasma cells, spatial heterogeneity of bone microbiome	Bone microbiota in DFO is polymicrobial, Gram-negative-enriched, and spatially heterogeneous	[9]
DFO (34)	Foot bone	**Dominant bacteria:** *Staphylococcus*, *Corynebacterium*, *Streptococcus*, *Propionibacterium*	Moderate-severe DFI ↑ DFO risk ↑ Bone infection likelihood	DFO exhibited a polymicrobial bone microbiota rather than a single-pathogen profile	[11]
DFO (20)	Foot bone	**Dominant genera:** *Corynebacterium*, *Finegoldia*, *Staphylococcus*, *Streptococcus*, *Porphyromonas*, *Anaerococcus*, Biofilm producing bacteria	Biofilm detected in ~ 80% of total bone samples	Bone microbiota in DFO is polymicrobial and biofilm-associated bacteria	[29]
DFO (17) *vs.* PFO (11)	Foot bone	↑ Alpha diversity, ↑ Firmicutes, ↑ Prevotellaceae, ↑ Prevotella, ↑ Gram-negative bacteria and anaerobes	↑ WBC, ↑ Neutrophil, ↑ Systemic inflammation (CRP, PCT, ESR) Infection duration ∝ *Prevotella* spp. Infection index ∝ *Proteus vulgaris* Infection index ∝ 1 / *Bacteroides fragilis*	Bone microbiota differs between DFO and PFO, with DFO showing higher diversity, anaerobic enrichment, and microbiome-clinical associations	[28]
COMJ; stages: I (7), stages: II (4), stages: III (5)	Jawbone	Stages II↓ Alpha diversity ↑ Oral/periodontal-associated bacteria *(Fusobacterium, Porphyromonas, Tannerella)* ↑ Anaerobic bacteria	Stage II↓ Lesions↑ Sequestration	Necrotic jaw bone lesions were enriched with anaerobic periodontal-associated pathobionts, suggesting adaptation to hypoxic bone environments	[30]

DFO: Diabetic foot osteomyelitis; PFO: posttraumatic foot osteomyelitis; DFI: diabetic foot infection; COMJ; chronic osteomyelitis of the jaw; WBC: white blood cells; CRP: C-reactive protein; PCT: procalcitonin; ESR: erythrocyte sedimentation rate; ↑: increased; ↓: decreased; ∝: associated with.

Several studies have reported an enrichment of Gram-negative anaerobic and biofilm-forming bacteria in osteomyelitic bone tissue^[28,29]^. The predominance of these microorganisms may contribute to the persistence of infection through multiple mechanisms. Gram-negative bacteria produce lipopolysaccharides (LPS), which activate inflammatory signaling pathways and stimulate the production of pro-inflammatory cytokines, thereby promoting osteoclastogenesis and bone resorption^[31,32]^. In addition, anaerobic bacteria are well adapted to the hypoxic microenvironment of infected bone and may facilitate the establishment of polymicrobial communities, enhancing bacterial survival and persistence. Biofilm-forming bacteria further contribute to chronic osteomyelitis by producing an extracellular polymeric matrix that protects embedded microorganisms from antimicrobial penetration and host immune clearance^[8]^. Biofilm formation also promotes bacterial adhesion to bone surfaces, facilitates interspecific cooperation, and enables the persistence of metabolically inactive bacterial populations that are less susceptible to antibiotic treatment^[8]^. Consequently, biofilm-associated infections are often characterized by recurrent inflammation, impaired bacterial eradication, and progressive bone destruction. Consistent with these mechanisms, Johani *et al.* reported a high abundance of biofilm-associated genera, including *Staphylococcus*, *Corynebacterium*, *Streptococcus*, *Finegoldia*, *Porphyromonas*, *Anaerococcus*, and *Propionibacterium*, in bone samples obtained from patients with DFO^[29]^. The detection of these taxa is consistent with a potential role for biofilm-associated communities in persistent DFO, although their presence alone does not establish viable colonization or their source of infection. Moreover, a comparative analysis of the bone microbiome between DFO and posttraumatic foot osteomyelitis (PFO) demonstrated increased microbial alpha diversity and enrichment of Gram-negative anaerobic bacteria in DFO^[28]^. These microbial alterations were associated with elevated systemic inflammatory markers, infection-related indices, and prolonged infection duration^[28]^. These findings indicate an association between the microbial profile of DFO and clinical features of infection and inflammation, although the cross-sectional design does not establish whether these microbial alterations contribute to disease progression.

Host receptor-mediated pathogen interactions may represent an additional mechanism contributing to the persistence of infectious osteomyelitis. Bacterial pathogens can exploit host cell-surface receptors to alter intracellular signaling, impair immune responses, and promote survival within the host. In particular, *Staphylococcus aureus* leukotoxins can interact with chemokine receptors, including CXCR1, CXCR2, and CCR5, on immune cells, while other staphylococcal leukotoxins exert cytolytic effects through atypical chemokine receptor 1 (ACKR1)^[33]^. These receptor-mediated interactions can disrupt immune-cell function and contribute to inflammation, immune evasion, and tissue injury. Such mechanisms may complement established pathways involving pattern-recognition receptors and biofilm-mediated immune escape in infectious osteomyelitis. However, the specific contribution of GPCR- and chemokine receptor-mediated pathogen interactions within the osteomyelitic bone microenvironment remains insufficiently defined and requires direct experimental validation.

Diabetes may further modify the bone microenvironment through immune dysregulation. Recent transcriptomic and single-cell analyses of diabetes-associated osteoporosis identified altered immune-cell profiles and enrichment of diabetes-related gene signatures in myeloid cells, including genes involved in inflammatory signaling such as TLR4, IL1R2, and IL4R^[34]^. Diabetes-associated immune changes, including an altered Treg/Th17 balance, macrophage polarization, and chronic inflammation, may favor a pro-osteoclastic and anti-osteogenic environment. Myeloid-cell signaling through inflammatory pathways, including TLR4/NF-κB and adipokine-associated pathways such as RESISTIN and VISFATIN, may further promote osteoclast activation and impair osteoblast function^[34]^. Although these observations were derived primarily from studies of diabetic osteoporosis rather than osteomyelitis, they provide a biological framework for understanding how diabetes may impair bone remodeling and host defense in diabetic foot osteomyelitis.

Evidence from chronic osteomyelitis of the jaw indicates a distinct local microbial profile in this subtype. Samples from necrotic jawbone lesions exhibited reduced microbial diversity and enrichment of anaerobic periodontal-associated bacteria, including *Fusobacterium*, *Porphyromonas*, and *Tannerella*^[30]^. The predominance of these taxa is consistent with their adaptation to the hypoxic conditions that develop during chronic infection. Interestingly, these microbial profiles differ from those reported in diabetic foot osteomyelitis, indicating that microbial communities detected in osteomyelitic bone may vary according to the anatomical site and disease context. Although similarities with microorganisms found in adjacent tissues may suggest a potential local source, current evidence does not establish direct transmission to bone.

Overall, the available evidence indicates that osteomyelitic bone contains distinct and frequently polymicrobial communities that are associated with clinical features of infection and inflammation. However, most evidence is derived from cross-sectional studies and does not establish whether microbial alterations are a cause or a consequence of chronic bone infection. Potential confounding factors, including prior antibiotic exposure, surgical history, diabetes, and other patient characteristics, may also influence the observed microbial profiles. Differences in sampling sites, sequencing methodologies, and patient populations may further contribute to the variability observed across studies. Future studies using standardized sampling strategies, longitudinal designs, strain-level tracking, viability assessment, and mechanistic approaches are needed to define the origin and functional significance of microorganisms detected in osteomyelitic bone.

## ORAL MICROBIOME AND OSTEOMYELITIS: CLINICAL EVIDENCE

Clinical studies suggest that osteomyelitis is associated with alterations in the oral microbiome, characterized by a reduction in microbial diversity and a shift in the abundance of specific oral taxa. These changes have been reported in both bacterial and nonbacterial forms of osteomyelitis, including chronic bacterial osteomyelitis (CBO) and CNO^[10,16,35]^. Importantly, oral microbial alterations have also been associated with host inflammation and immune-related parameters, suggesting a potential link between oral dysbiosis and osteomyelitis pathogenesis as summarized in Table 3.

**Table 3:** Oral microbiomes in osteomyelitis: clinical studies

**Model (N)**	**Bone site**	**Oral microbiome**				**Other parameters**	**Interpretation**	**Ref**
		**Oral sampling site**	**α diversity**	**β diversity**	**Microbiota alteration**			
CBO (5) *vs.* CNO (10) *vs.* HC (5)	Jawbone	Saliva	NA	NA	*Mogibacterium*: CBO > CNO ≈ HC	NA	*Mogibacterium* was enriched in chronic bacterial osteomyelitis compared with CNO and healthy controls	[16]
CNO (20) *vs.* HC (36)	Multiple bones (long bones, clavicle, mandible, vertebrae)	Tongue swab (tongue center and left tongue)	**↓**	Difference	**Site-specific bacteria Left tongue:** ↑ *Megasphaera*, ↑ *Haemophilus*, ↑ *Lachnoanaerobaculum*, ↑ *Corynebacterium* **Tongue center:** ↑ *Prevotella*, ↑ *Fusobacterium*, ↑ *Porphyromonas*, ↑ *Mogibacterium*, ↑ *Actinomyces* spp.	Age, medication exposure, and oral sampling site ∝ microbiome variation	Oral microbiome variation in CNO was associated with age, medication exposure, and oral sampling site	[35]
CNO (25) *vs.* HC (24)	Multiple bones (long bones, clavicle, mandible, vertebrae)	Saliva	**↓**	Difference	↑ HACEK group (*Eikenella*, *Kingella*, *Cardiobacterium*), ↑ Pathobionts (*Actinomyces*, *Leptotrichia*, *Campylobacter*, *Corynebacterium* / *Propionibacteriaceae*)	MCHC ∝ *Kingella*, *Cardiobacterium* Lymphocyte number ∝ *Campylobacter* LDH activity ∝ uncl. *Prevotellaceae*	The oral microbiome, in particular the enrichment of pathobionts, is associated with host immune and physiological parameters linked to bone inflammation	[10]

CBO: Chronic bacterial osteomyelitis; CNO: chronic nonbacterial osteomyelitis; HC: healthy controls; HACEK: *Haemophilus, Aggregatibacter, Cardiobacterium, Eikenella,* and *Kingella*; MCHC: mean corpuscular hemoglobin concentration; LDH: lactate dehydrogenase; NA: not reported; ↑: increased; ↓: decreased; ∝: associated with; >: higher than; ≈: approximately equal to.

CBO is characterized by persistent bacterial infection and chronic inflammation within bone tissue. Yahara *et al.* compared the oral microbiomes of patients with jawbone CBO, CNO, and healthy controls^[16]^. The authors reported a significantly higher abundance of *Mogibacterium* in patients with CBO than in those with CNO or healthy controls^[16]^. *Mogibacterium* is a strict anaerobe frequently detected in periodontal infections and oral biofilms^[13,36]^. Its enrichment may indicate a shift toward an anaerobic microbial community in CBO. In addition, increased oral abundance of *Mogibacterium* has been associated with the progression of medication-related osteonecrosis of the jaw (MRONJ) and has shown a correlation with elevated levels of pro-inflammatory cytokines, including IFN-γ and TNF-α^[37]^. These findings suggest a potential association between *Mogibacterium* and the pro-inflammatory environment of jawbone lesions, although its direct contribution to persistent inflammation has not been established.

CNO is an autoinflammatory bone disease characterized by sterile bone inflammation without evidence of active bacterial infection^[7,38]^. Immune dysregulation is considered a central mechanism underlying this disease^[38]^. Alterations in the oral microbiome have been reported in patients with CNO, including a reduction in microbial diversity and the enrichment of several oral taxa associated with inflammatory conditions^[10,35]^. Zeus *et al.* examined the tongue microbiome of patients with CNO and identified site-specific microbial alterations in both the central and lateral regions of the tongue in comparison with healthy controls^[35]^. These microbial differences were associated with age and exposure to medication^[10,35]^. These findings also suggest that age and medication exposure may influence the oral microbiome and should be considered when interpreting disease-associated microbial alterations. Similarly, Rausch *et al.* reported enrichment of HACEK group members, including *Eikenella*, *Kingella*, and *Cardiobacterium*, together with increased abundance of several oral pathobionts^[10]^. Notably, these microbial changes were associated with host physiological and immune parameters, including mean corpuscular hemoglobin concentration and lymphocyte count^[10]^. The enrichment of HACEK members and oral pathobionts may reflect increased exposure to microbial-associated molecular patterns capable of stimulating innate immune responses^[39,40]^. Persistent activation of these pathways can promote the production of pro-inflammatory cytokines, including TNF-α, IL-1β, and IL-6, which are known to contribute to osteoclast activation and bone inflammation^[41,42]^. Although a causal relationship has not been established, these observations raise the possibility that oral microbial dysbiosis may influence the inflammatory environment associated with CNO. Oral microbial dysbiosis has been reported in both CBO and CNO, but the microbial profiles differ between the two conditions. CBO is characterized by an enrichment of anaerobic bacteria in association with chronic infection, whereas CNO is associated with microbial alterations linked to immune and inflammatory responses. These findings are consistent with the distinct pathogenic mechanisms underlying infectious and autoinflammatory bone diseases.

The oral microbiome appears to be associated with both microbial persistence and host inflammatory responses in osteomyelitis. However, current evidence is primarily derived from observational studies and does not establish whether oral dysbiosis contributes directly to disease pathogenesis or develops secondary to chronic inflammation and antimicrobial treatment. Potential confounding factors, including age, medication use, and prior antibiotic exposure, should also be considered when interpreting these associations. Future longitudinal and mechanistic studies are required to clarify the role of the oral microbiome in osteomyelitis.

## GUT MICROBIOME AND OSTEOMYELITIS: CLINICAL AND PRECLINICAL EVIDENCE

Osteomyelitis has traditionally been considered a localized bone infection. However, there is accumulating evidence to suggest that alterations in the gut microbiota may be associated with osteomyelitis and may influence systemic immune and inflammatory responses^[10,23,43]^. In a study of patients with CNO, fecal microbial alpha diversity did not differ significantly from that of healthy controls, although the overall microbial community composition differed between the groups^[10]^. Several genera, including *Barnesiella*, *Christensenella*, *Paraprevotella*, and *Coprobacter*, were less abundant in patients with CNO, whereas *Faecalibacterium* was associated with CNO [Table 4]^[10]^. However, the clinical significance of these microbial alterations remains unclear. Therefore, the available evidence suggests an association between CNO and alterations in gut microbial composition but does not establish whether these changes are related to disease activity or contribute to disease pathogenesis. Previous studies have reported that *Christensenella* and *Paraprevotella* are commensal gut bacteria associated with the production of short-chain fatty acids and immune regulation^[44,45]^. Therefore, their depletion may reflect disruption of gut microbial homeostasis and immune balance in CNO. The observed association with *Faecalibacterium* should also be interpreted cautiously, as this genus is generally considered a beneficial butyrate-producing bacterium^[46]^. These findings indicate alterations in gut microbial composition in CNO, but their relationship with disease activity and chronic bone inflammation remains unclear.

**Table 4:** Gut microbiomes in osteomyelitis: clinical study

**Model (N)**	**Bone site**	**Gut Microbiome**			**Interpretation**	**Ref**
		**α diversity**	**β diversity**	**Microbiota alteration**		
CNO (25) *vs.* HC (24)	Multiple bones (long bones, clavicle, mandible, vertebrae)	↔	Difference	**↑** *Faecalibacterium*, **↓** *Barnesiella*, **↓** *Christensenella*, **↓** *Paraprevotella*, **↓** *Coprobacter*	Gut microbial composition differed between patients with CNO and healthy controls	[10]

**Table 5:** Gut microbiome in osteomyelitis: animal studies

**OM Type**	**Animal Model**	**Induction of Osteomyelitis / location**	**Disease severity**	**Major findings**			**Other findings**	**Interpretation**	**Ref**
				**α diversity**	**β diversity**	**Alterations of gut microbiota**			
TO	SD rats	LPS, 2.56 mg/kg / Drilling tibia	++	↓	Difference	↑ *Blautia* ↑ *Fusicatenibacter* ↑ *Enterococcus* ↓ *Dubosiella* ↓ *Prevotella* ↓ *Bacillus*	**Bone Repair** ↓ BMD ↓ BV/TV ↓ Tb.N ↓ Tb.Sp **Inflammation** ↑ Serum TNF- ↑ Serum IL-6 **Serum SCFAs** ↑ Acetic acid ↑ Isobutyric acid ↑ Isovaleric acid ↑ Caproic acid ↔ Propionic acid ↔ Butyric acid ↔ Valeric acid ↔ 4-Methylvaleric acid	TO was characterized by impaired bone repair, elevated inflammatory cytokines, gut microbial dysbiosis, and altered SCFA profiles	[23]
BCO	Chickens(D1, 17, 56)	Wire-flooring stress / Femur, Tibia	D56 > D17 > D1	D56 > D17 > D1	Difference	D56: ↑ *Ruminococcus*	**Lameness:** D56 > D17 > D1 **Bone lesions:** D56 > D17 > D1 **Stress:** D56 > D17 > D1 **Shared gut–bone taxa: ** *Ruminococcus*, *Bacillus*, *Faecalibacterium*, *Blautia*, *Actinomyces*	Gut microbiota composition changed during BCO progression, with potential gut-to-bone bacterial translocation	[43]

CNO: Chronic nonbacterial osteomyelitis; HC: healthy controls; α: alpha diversity; β: beta diversity; ↑: increased; ↓: decreased.

Evidence from animal studies further supports a link between gut microbiota alterations and experimental models of osteomyelitis [Table 5]. Zhou *et al.* established a rat model of traumatic osteomyelitis (TO) by tibial drilling followed by administration of LPS^[23]^. TO was associated with gut microbial dysbiosis, characterized by increased abundances of *Blautia*, *Fusicatenibacter*, and *Enterococcus*, together with decreased abundances of *Dubosiella*, *Prevotella*, and *Bacillus*^[23]^. These microbial alterations were accompanied by elevated levels of serum TNF-α and IL-6 and impaired bone repair^[23]^. The enrichment of *Enterococcus* together with the depletion of several commensal bacteria may be associated with systemic inflammatory responses. TNF-α and IL-6 are known to suppress osteoblast activity and promote osteoclastogenesis, thereby impairing bone regeneration^[47]^. Although these findings suggest a potential relationship among gut dysbiosis, systemic inflammation, and impaired bone repair in this experimental model, the LPS-induced model does not fully reproduce the pathophysiology of human infectious osteomyelitis.

Bacterial chondronecrosis with osteomyelitis (BCO) is a common bone disease in broiler chickens that primarily affects the femur and tibia^[48,49]^. In a wire-flooring stress-induced BCO model, disease progression was associated with alterations in the composition of the gut microbiota ^[43]^. Several bacterial genera, including *Ruminococcus*, *Bacillus*, *Faecalibacterium*, *Blautia*, and *Actinomyces*, were identified in both gut and bone samples^[43]^. One proposed mechanism is that gut dysbiosis may increase intestinal permeability and facilitate the translocation of bacteria from the gut into the circulation. Circulating bacteria or bacterial products may then reach mechanically stressed regions of the femur and tibia, where vascular damage and local hypoxia create a favorable niche for bacterial colonization. This process may amplify local inflammation, impair bone integrity, and contribute to chondronecrosis and osteomyelitis development. However, the detection of the same bacterial genera in gut and bone samples does not establish direct bacterial translocation or a common strain origin. These findings support a potential association between changes in the gut microbiota and bone lesions in the BCO model. Overall, the available evidence supports an association between gut microbial alterations, systemic inflammation, and bone pathology in experimental models of osteomyelitis. However, most evidence is derived from experimental animal models, whereas clinical studies remain limited. The available models also differ substantially in disease etiology, host species, and experimental design and should not be directly extrapolated to human infectious osteomyelitis. In addition, the mechanisms linking gut dysbiosis to bone infection have not been fully elucidated. Future studies integrating longitudinal clinical cohorts with mechanistic investigations are needed to establish causal microbiome-host interactions and evaluate the therapeutic potential of targeting the gut microbiome in osteomyelitis.

## INTEGRATION OF MICROBIOME-BONE AXIS IN OSTEOMYELITIS

The skin, oral, bone, and gut microbiomes occupy distinct anatomical niches. However, the available evidence suggests that they may be associated with osteomyelitis through different local and systemic pathways [Figure 1]. The skin and oral microbiomes represent potential reservoirs of microorganisms associated with osteomyelitis. Microbial taxa detected in infected bone may overlap with those commonly found at these anatomical sites; however, such overlap does not establish their origin or demonstrate direct transmission to bone. Current studies generally rely on microbial detection and lack strain-level tracking, assessment of microbial viability, and longitudinal sampling. Therefore, detection of these microorganisms in bone tissue should be distinguished from viable colonization and direct evidence of their source. Once infection is established, microorganisms present within bone tissue may form polymicrobial communities and biofilms that contribute to bacterial persistence and reduced susceptibility to host immune clearance and antimicrobial therapy^[11,28,29]^. These processes may favor persistent infection and chronic inflammation.

**Figure 1:**
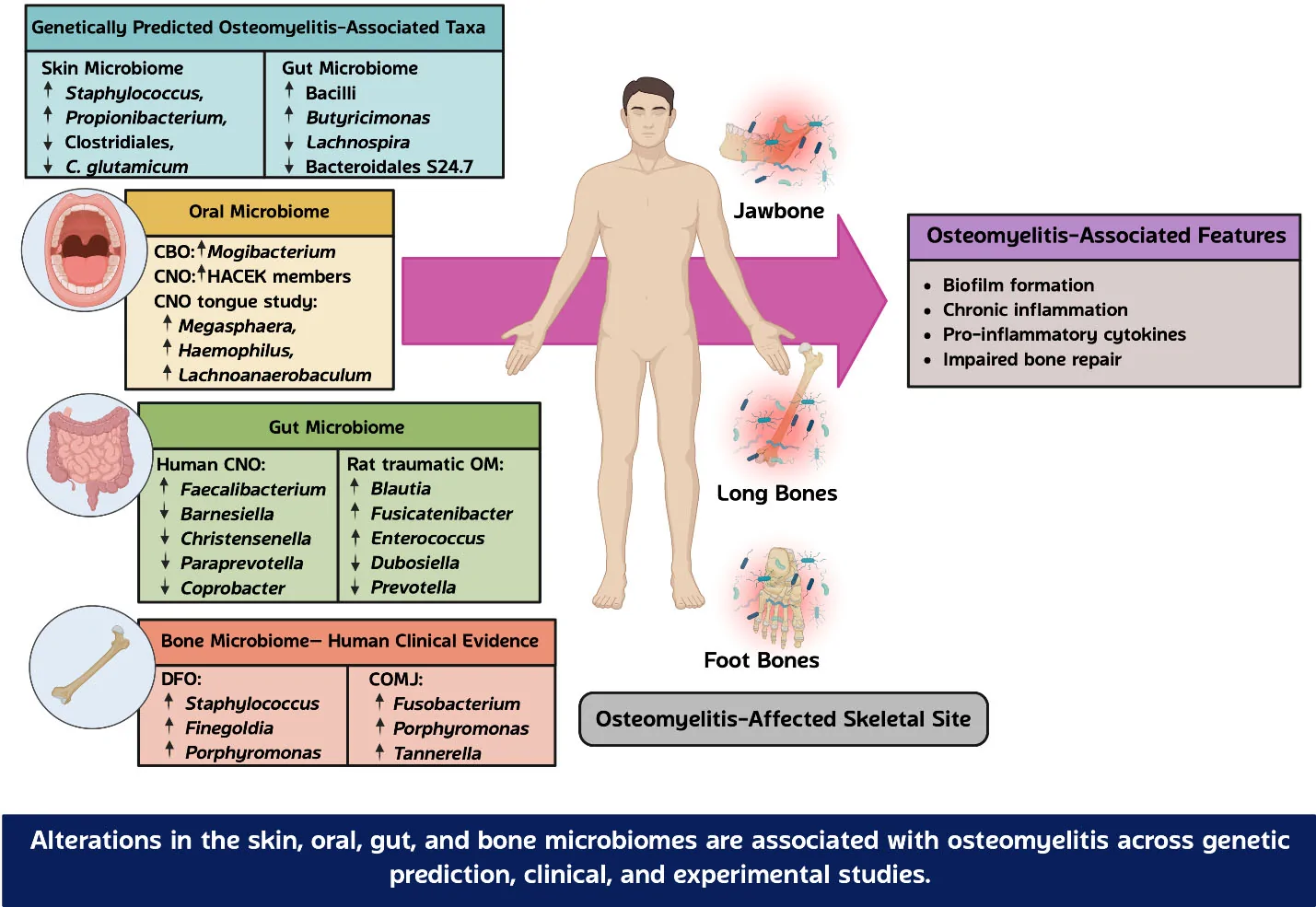
Overview of microbiome alterations associated with osteomyelitis. Alterations in the skin, oral, gut, and bone microbiomes have been associated with osteomyelitis across genetic-prediction, clinical, and experimental studies. Genetic-prediction studies have identified skin- and gut microbiota-associated taxa linked to osteomyelitis susceptibility. Clinical studies have reported microbial profiles or alterations in the oral, gut, and bone microbiomes in different forms of osteomyelitis, whereas experimental studies have demonstrated gut microbiome alterations in animal models. These microbiome alterations are associated with osteomyelitis-related features, including biofilm formation, chronic inflammation, pro-inflammatory cytokine production, and impaired bone repair. Representative microbial taxa and their directions of association or alteration are shown. Created with BioRender. CBO: Chronic bacterial osteomyelitis; CNO: chronic nonbacterial osteomyelitis; OM: osteomyelitis; DFO: diabetic foot osteomyelitis; COMJ: chronic osteomyelitis of the jaw. Created in BioRender.

Within the infected bone microenvironment, microbial persistence is accompanied by sustained activation of the host immune response. Bacterial components, including lipopolysaccharides, lipoteichoic acid, peptidoglycan, and other microbial-associated molecular patterns, activate pattern-recognition receptors on immune and bone cells^[32]^. Subsequent activation of NF-κB and related inflammatory signaling pathways stimulates the production of pro-inflammatory cytokines, including TNF-α, IL-1β, and IL-6^[2,32]^. Persistent inflammatory signaling promotes osteoclast differentiation through the RANK/RANKL pathway while suppressing osteoblast function, resulting in progressive resorption of the bone, impaired bone formation, and delayed skeletal repair. In parallel, biofilm-associated bacteria and intracellular pathogens evade immune surveillance, allowing inflammation to persist despite antimicrobial treatment^[8]^.

In contrast to the skin, oral cavity, and bone, the gut microbiome is unlikely to serve as a direct source of bone pathogens. Instead, a potential contribution of the gut microbiome may involve systemic regulation of host immunity and bone metabolism. Gut microbial dysbiosis can impair the integrity of the intestinal barrier, allowing microbial products and metabolites to enter the circulation and influence distant tissues. Altered production of SCFAs, together with increases in systemic inflammatory mediators, may disturb immune homeostasis, enhance osteoclastogenesis, and reduce osteoblast activity^[14]^. These mechanisms provide a potential biological link between gut dysbiosis and bone homeostasis; however, their direct contribution to osteomyelitis in humans remains unclear.

The systemic effects of the gut microbiota may extend beyond direct gut-bone communication. Gut-derived microbial products and metabolites can enter the circulation and influence immune and metabolic responses in distant organs. In autoimmune and inflammatory diseases, impaired intestinal barrier integrity, microbial translocation, and alterations in SCFAs, tryptophan metabolites, and bile acid metabolites have been linked to systemic immune dysregulation^[50]^. Emerging evidence also supports a gut-brain-bone axis, in which gut-derived metabolites and immune mediators interact with neuroendocrine pathways that regulate skeletal remodeling^[51]^. Conversely, neural and stress-related signals may alter gut function and microbial composition, indicating bidirectional communication among the gut, nervous system, immune system, and bone. Although these pathways have been studied mainly in inflammatory and metabolic bone disorders, they provide a broader biological framework for understanding how gut microbial alterations could influence systemic inflammation and bone homeostasis. Their relevance to osteomyelitis, however, remains to be established.

Current evidence therefore supports a conceptual framework in which local and systemic microbiomes may be associated with osteomyelitis through distinct but potentially complementary pathways. The skin, oral, and bone microbiomes are primarily associated with local microbial communities, biofilm formation, and inflammatory responses, whereas the gut microbiome may influence systemic immunity, inflammatory tone, and bone remodeling. Although the contribution of each microbial niche differs, these local and systemic pathways may collectively shape the inflammatory and skeletal environment associated with osteomyelitis. This integrated microbiome-bone axis provides a framework for understanding potential interactions between microbial alterations, host inflammation, and bone remodeling and may guide future investigation of microbiome-targeted strategies for the prevention and treatment of osteomyelitis.

## MICROBIOME-TARGETED INTERVENTIONS FOR OSTEOMYELITIS

Previous GWAS-based MR analyses, clinical studies, and preclinical studies have identified associations between alterations in the skin, oral, bone, and gut microbiomes and osteomyelitis. These findings suggest that the microbiome may play a role in the pathogenesis of osteomyelitis and highlight its potential as a therapeutic target. Accordingly, several microbiome-targeted interventions have been investigated in experimental models of osteomyelitis and related bone disorders, as summarized in Table 6 and Figure 2.

**Figure 2:**
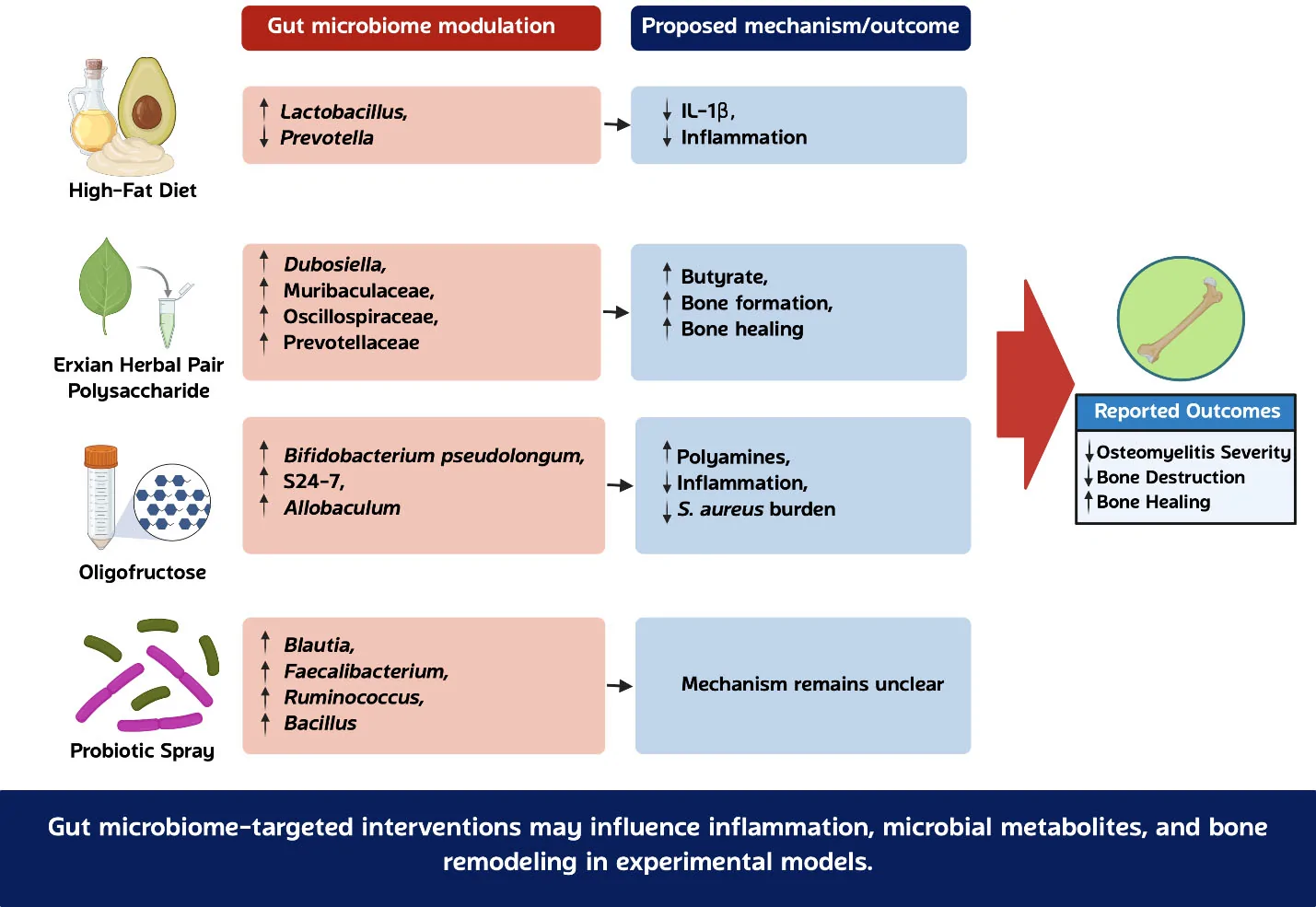
Microbiome-targeted interventions investigated in experimental models of osteomyelitis and related bone disorders. High-fat diet, Erxian Herbal Pair polysaccharide, oligofructose, and probiotic supplementation have been associated with distinct alterations in the gut microbiota and related inflammatory, metabolic, and bone outcomes. These interventions have been investigated across different experimental models and should not be considered equivalent or directly extrapolated to human osteomyelitis. Arrows indicate the reported direction of change. Created with BioRender. IL: Interleukin. Created in BioRender.

**Table 6:** Microbiome-targeted interventions for osteomyelitis: animal studies

**Animal model**	**OM induction/ site**	**Interventions/Duration/N**	**Severity of OM**	**Major findings**			**Other findings**	**Interpretation**	**Ref**
				**α diversity**	**β diversity**	**Alterations of gut microbiota**			
Male BALB/cJ *Pstpip2^cmo^* mice	Spontaneous CMO (Pstpip2 mutation)	HFD (40% fat)/100 days/22 *vs.* LFD (5% fat)/100 days/40	↓	NA	Difference	↑ *Lactobacillus* ↓ *Prevotella*	↓ Osteomyelitis incidence, ↓ Bone erosion, ↓ Inflammatory cell infiltration, ↓ IL-1β expression	HFD-mediated modulation of gut microbiota reduced severity and inflammation of osteomyelitis	[21]
Obese maleC57BL/6J mice with T2DM Lean male C57BL/6J mice	*S. aureus* USA300 coated pin / Tibia	Oligofructose /4 weeks/13 *vs.* Cellulose/4 weeks/13 Oligofructose /4 weeks/13 *vs.* Lean fed with Cellulose/4 weeks/13	++ +++ + ++	↓	Difference	**Oligofructose group** ↑ *Bifidobacterium pseudolongum* ↑ *S24-7 family* ↑ *Allobaculum*	↓ Tibial *S. aureus* burden↓ Soft tissue abscess burden ↓ Abscess size ↓ TNF-α ↓ IL-6 ↑ Polyamines (spermine, spermidine)	Oligofructose-mediated modulation of gut microbiota and polyamine metabolism reduced osteomyelitis severity in obese/T2D mice	[22]
SD rats	LPS, 2.56 mg/kg / Drilling tibia	EHP(300 mg/kg/day)/4 weeks/12 *vs.* TO model (saline)/4 weeks/12	↓	↑	Difference	↑ *Dubosiella*, ↑ *Prevotella*, ↑ Muribaculaceae, ↑ Oscillospiraceae, ↑ Prevotellaceae	↑ BMD, ↑ BV/TV, ↑ ALP activity, ↑ Butyrate production ↓ Inflammatory cell infiltration	EHP promoted bone repair through modulation of gut microbiota and butyrate production	[23]
Chickens	Wire-flooring stress / Femur, Tibia	*Bacillus*-base probiotic sprays (*E. faecium*, *B. amyloliquefaciens* 516, *B. subtilis* 597, *B. subtilis* 600)/56 days/50 *vs.* One probiotic spray (*E. faecium*)/56 days/50 *vs.* BCO model /56 days/50	+ ++ +++	↔	NS	*Bacillus*-base Probiotic ↑ *Blautia*, ↑ *Faecalibacterium*, ↑ *Ruminococcus*, ↑ *Bacillus*, ↑ *Actinomyces*	**Lameness incidence** Four probiotic < One probiotic < Control	*Bacillus*-based probiotic reduced BCO-associated lameness despite minimal changes in overall microbiota diversity	[24]
C57BL/6 mice	*S. aureus* solution injection / femur	High dose antibiotic cocktail/5 weeks/10 *vs.* CO model (PBS)/5 weeks/10	↑	↓	Difference	↓ *Bacteroidetes* ↓ *Akkermansia* ↓ *Roseburia* ↓ *Bifidobacterium* ↑ *Proteobacteria*	↑ Bacterial burden in bone ↓ Survival	Antibiotic-induced gut dysbiosis was associated with increased bacterial burden and reduced survival in mice with chronic osteomyelitis	[25]
C57BL/6 mice	No osteomyelitis / intramedullary femoral implant	High-dose antibiotic cocktail / 5 weeks / 10 *vs.* implant model/5 weeks/10	NA	↓	Different	↓ Bacteroidetes, ↓ *Akkermansia*, ↓ *Roseburia*, ↓ *Bifidobacterium*, ↑ Proteobacteria	↓ BMD, ↓ BV/TV, ↓ Osseointegration, ↓ Osteoblastogenesis, ↑ Osteoclastogenesis, ↑ TNF-α, ↑ IL-6	Antibiotic-induced gut dysbiosis impaired osseointegration and bone remodeling and increased systemic inflammation in an intramedullary implant model without osteomyelitis	[25]

ALP: Alkaline phosphatase; BCO: bacterial chondronecrosis with osteomyelitis; BMD: bone mineral density; BV/TV: bone volume/tissue volume; CMO: chronic multifocal osteomyelitis; CO: chronic osteomyelitis; EHP: Erxian herbal pair polysaccharide; HFD: high-fat diet; IL: interleukin; LFD: low-fat diet; NA: not available; NS: not significant; OM: osteomyelitis; PBS: phosphate-buffered saline; Pstpip2: proline-serine-threonine phosphatase interacting protein 2; T2DM: type 2 diabetes mellitus; TNF: tumor necrosis factor; TO: traumatic osteomyelitis; α: alpha diversity; β: beta diversity; ↑: increased; ↓: decreased; ↔: no significant change; <: lower than; >: greater than.

Dietary modulation of the gut microbiota has been investigated as a potential strategy for modifying disease severity in experimental CNO. In a *Pstpip2^cmo^* mouse model of CNO, a high-fat diet reduced disease severity, inflammatory cytokine production, and bone destruction^[21]^. These beneficial effects were accompanied by alterations in the composition of the gut microbiota, including a reduction in *Prevotella* abundance and an increased abundance of *Lactobacillus*^[21]^. Previous studies have shown that *Prevotella* can activate Toll-like receptor 2 (TLR2), leading to the production of Th17-polarizing cytokines, including IL-23 and IL-1^[52,53]^. In addition, *Prevotella* can stimulate epithelial cells to produce IL-8, IL-6, and CCL20, thereby promoting Th17 responses and neutrophil recruitment^[52]^. Conversely, it has been reported that Lactobacillus species suppress inflammatory responses by the downregulation of Th17 cells and the reduction of the production of pro-inflammatory cytokines, including IL-17F and TNF-α^[54-56]^. Therefore, the reduction in Prevotella and enrichment of *Lactobacillus* may have contributed to the attenuation of inflammation and protection against bone destruction observed in this model. These findings suggest a potential relationship between diet-induced changes in the gut microbiota and reduced inflammation in this CNO model. However, CNO is an autoinflammatory rather than infectious bone disease, and the effects of a high-fat diet in this model should not be extrapolated to human infectious osteomyelitis.

Oligofructose, a fermentable dietary fiber, has also been investigated as a microbiome-targeted intervention in experimental infectious osteomyelitis. In an obesity-associated type 2 diabetes mouse model of *Staphylococcus aureus*-infected osteomyelitis, supplementation with oligofructose reduced the bacterial burden in bone and soft tissue and attenuated systemic inflammation^[22]^. These effects were accompanied by marked alterations in the composition of the gut microbiota, including an increased abundance of *Bifidobacterium pseudolongum*^[22]^. Metabolomic analysis further demonstrated increased production of microbiota-derived polyamines, particularly spermidine and spermine^[22]^. Notably, direct polyamine supplementation produced similar beneficial effects^[22]^. Previous studies have shown that polyamines can reduce inflammation by suppressing the production of pro-inflammatory cytokines, including TNF-α, IL-6, and IL-1β^[57,58]^. Polyamines may influence bone remodeling through several mechanisms. Alterations in polyamine metabolism have been associated with osteoblastogenesis^[59]^, while spermidine and spermine have been reported to prevent bone loss by inhibiting osteoclast differentiation and maturation^[60]^. In addition, exogenous polyamines can promote osteogenic differentiation of hBMSCs^[61]^. These findings suggest that microbiota-derived polyamines may contribute to both inflammation control and bone healing in this experimental model.

Recent evidence suggests that microbiome-targeted polysaccharides may represent a potential approach for modulating bone repair in experimental osteomyelitis. In a rat model of traumatic osteomyelitis, administration of polysaccharides isolated from the Erxian herbal pair improved bone healing, as demonstrated by increases in bone mineral density, trabecular bone formation, and alkaline phosphatase activity^[23]^. Polysaccharide treatment altered the gut microbiota and increased the abundance of several beneficial taxa associated with SCFA production, including *Muribaculaceae*, *Prevotella*, and *Oscillospiraceae*^[23]^. These microbial alterations were accompanied by the elevation of butyrate concentrations in both feces and serum^[23]^. Further *in vitro* experiments demonstrated that butyrate enhanced osteoblast activity and suppressed the MEK/ERK/MAPK signaling pathway, suggesting a potential mechanism linking gut microbial metabolites to bone regeneration^[23]^. Together, these findings support a potential gut microbiota-butyrate-bone pathway in this experimental setting. However, the traumatic LPS-induced rat model does not fully reproduce human infectious osteomyelitis.

In a broiler model of BCO, administration of *Bacillus*- and *Enterococcus*-based probiotics significantly reduced BCO-associated lameness^[24]^. Probiotic supplementation was associated with increased abundance of several bacterial genera, including *Blautia*, *Faecalibacterium*, *Ruminococcus*, *Bacillus*, and *Actinomyces*^[24]^. However, no substantial differences in cecal microbial diversity or overall community composition were observed between treatment groups^[24]^. These findings suggest that probiotic supplementation may provide protection against osteomyelitis, although the underlying mechanisms remain unclear.

In contrast to prebiotic and probiotic approaches, antibiotic-induced disruption of the gut microbiota may adversely affect bone infection and osseointegration. In a mouse study, broad-spectrum antibiotic treatment markedly altered the gut microbiota and reduced several SCFA-producing or beneficial bacteria, including *Akkermansia*, *Bifidobacterium*, and *Roseburia*^[25]^. In mice with chronic osteomyelitis, antibiotic-treated mice showed a higher bacterial burden in bone and lower survival than untreated osteomyelitis mice^[25]^. However, no significant differences in bone mineral density or bone formation were observed between these groups. In the intramedullary implant model without osteomyelitis, antibiotic-treated mice showed reduced bone mass and bone formation, reduced osteoblast activity, and increased osteoclast activity compared with the implant group^[25]^. Previous studies have shown that SCFAs can regulate bone remodeling by suppressing osteoclast activity and promoting bone formation^[12,14,62]^. In addition, it has been reported that SCFAs influence bone metabolism through the regulation of the production of IGF-1 and the expansion of regulatory T cells, which support osteoblast function and inhibit osteoclastogenesis^[15,62]^. These findings suggest that antibiotic-induced gut dysbiosis may affect bone infection and bone remodeling through different pathways. However, the available evidence does not establish a direct mechanistic link between the observed microbial alterations and these outcomes.

Across these experimental studies, microbiome-targeted interventions were associated with changes in inflammation, microbial metabolites, or bone-related outcomes, although the effects varied according to the intervention and experimental model. These findings provide preliminary evidence that modulation of the gut microbiota may influence bone infection or remodeling under specific experimental conditions. However, the interventions have been evaluated in distinct disease models, including CNO, infectious osteomyelitis, traumatic LPS-induced osteomyelitis, and BCO, and should not be considered equivalent or directly extrapolated to human osteomyelitis. Their efficacy and safety in patients with osteomyelitis remain largely unknown. Future clinical studies are required to determine whether microbiome-targeted interventions can be translated into effective adjunctive therapies for osteomyelitis.

## CHALLENGES IN TRANSLATIONAL APPLICATION

Although microbiome-targeted interventions have shown beneficial effects in experimental models of osteomyelitis, their clinical application remains limited. Most evidence is currently derived from animal studies, and differences between animal models and humans may affect treatment responses. In particular, the reduced severity of CNO observed in mice fed a high-fat diet should not be interpreted as supporting the use of a high-fat diet in patients. Further studies are needed to determine the mechanisms underlying this finding and its clinical relevance.

Several challenges should also be considered before microbiome-targeted interventions can be translated into clinical practice. The optimal dose, duration, and composition of probiotics, prebiotics, and other microbiome-targeted interventions have not been established. Their efficacy and long-term safety in patients with osteomyelitis also remain unclear. Long-term adherence may also be challenging, and safety requires particular consideration in patients with diabetes or immunocompromised conditions. In addition, treatment responses may vary according to the baseline gut microbiota, underlying disease, and concurrent antibiotic treatment, which may complicate the standardization of microbiome-targeted therapies. Importantly, antibiotic therapy remains essential for the treatment of infectious osteomyelitis. Although prolonged antibiotic treatment may alter the gut microbiota and reduce SCFA-producing bacteria, preservation of the gut microbiota should not compromise effective antimicrobial treatment. Future studies should investigate strategies to reduce antibiotic-associated gut dysbiosis while maintaining adequate control of bone infection. Clinical trials are needed to determine the safety and efficacy of microbiome-targeted interventions in patients with osteomyelitis.

## LIMITATIONS, CONFOUNDING FACTORS, AND CAUSAL INFERENCE

Most clinical microbiome studies in osteomyelitis are cross-sectional and therefore cannot determine whether microbial alterations contribute to disease development or occur as a consequence of infection and inflammation. Several factors may also affect microbiota composition independently of osteomyelitis, including prior antibiotic exposure, surgical procedures, dietary patterns, diabetes, other underlying diseases, and differences in patient characteristics. These factors are particularly important in diabetic foot osteomyelitis, in which metabolic abnormalities, chronic wounds, repeated antibiotic exposure, and surgical treatment may influence the observed microbial profiles. Therefore, associations between microbial taxa and osteomyelitis should be interpreted cautiously and do not establish a direct causal relationship.

Differences in microbiome methodology may also contribute to variation among studies. Sampling sites and procedures, sample handling, DNA extraction methods, sequencing platforms, and sequencing batch effects can influence microbial profiles. Most available studies also rely on 16S rRNA gene sequencing, which mainly provides information on microbial composition and has limited resolution at the species or strain level. In addition, 16S rRNA sequencing does not directly determine microbial metabolic activity or virulence factor expression. The detection of taxa known to produce specific metabolites therefore does not confirm that these metabolites are altered. For example, the presence of SCFA-producing bacteria does not provide direct evidence of altered SCFA production without metabolomic measurements. Such data remain limited in patients with osteomyelitis. Future studies integrating shotgun metagenomics, metabolomics, transcriptomics, and other functional approaches are needed to determine the biological relevance of the observed microbial alterations.

Genetic prediction studies also have important limitations. Although Mendelian randomization can reduce conventional confounding and reverse causation, its interpretation depends on the validity of the genetic instruments and the assumptions of the analysis. Horizontal pleiotropy may influence the observed associations when genetic variants affect osteomyelitis through pathways unrelated to the microbial taxa of interest. In addition, most available GWAS-MR evidence is derived from the UK Biobank, which may limit its generalizability to populations with different ancestry, age, and metabolic conditions. Therefore, evidence from genetic prediction studies, clinical microbiome studies, and experimental models should be interpreted according to the strengths and limitations of each approach. Longitudinal studies using standardized microbiome methods and integrated functional analyses are needed to better define the causal relationship between microbiome alterations and osteomyelitis.

## FUTURE PERSPECTIVE AND CONCLUSIONS

Most studies have examined the skin, oral, bone, and gut microbiomes separately, despite growing evidence that these microbial communities are interconnected. Whether microbial alterations at one anatomical site influence the microbial composition and inflammatory responses at another site remains unclear. Simultaneous characterization of multiple microbial niches within the same patient may provide a more comprehensive understanding of microbiome-host interactions during osteomyelitis.

The current literature is also dominated by cross-sectional studies, making it difficult to distinguish microbial changes that contribute to disease development from those that occur secondarily to infection and inflammation. Longitudinal clinical studies and mechanistic experimental models are needed to define the temporal relationship between microbiome alterations, immune activation, and bone remodeling. Such studies may also clarify how microbial metabolites, biofilm formation, and host immune responses interact during disease progression.

Most microbiome studies in osteomyelitis have relied on 16S rRNA sequencing, which provides limited information on microbial function. Future studies should incorporate shotgun metagenomic sequencing together with metabolomics and host transcriptomic analyses to characterize microbial function and host responses. Integration of these approaches with clinical phenotypes may facilitate the identification of biomarkers associated with disease severity, prognosis, and treatment response.

Evidence supporting microbiome-targeted interventions remains largely preclinical. Well-designed clinical studies are needed to determine whether microbiome modulation can improve outcomes in patients with osteomyelitis. Defining the interactions among microbial communities, host immunity, and bone remodeling may provide a stronger foundation for the development of microbiome-based therapeutic strategies. Collectively, the available evidence supports a role for the microbiome in osteomyelitis pathogenesis through interconnected local and systemic mechanisms. Continued integration of clinical and mechanistic studies will be essential to translate these findings into microbiome-based strategies for the prevention and treatment of osteomyelitis.

## DECLARATIONS

### AI and AI-assisted tools statement

During the preparation of this manuscript, the AI tool Grammarly (version 1.2.263.18.93, released 2026-05-25) was used solely for language editing. The tool did not influence the study design, data collection, analysis, interpretation, or the scientific content of the work. All authors take full responsibility for the accuracy, integrity, and final content of the manuscript.

### Copyright

© The Author(s) 2026.
