# Supplementary material for: The microbiome bone axis in osteomyelitis: integrating evidence from bone oral and gut microbiota
Source: Microbiome Res Rep. 2026 Sep 29;5(3):23. doi: 10.20517/mrr.2026.63 (PMC13678545; doi:10.20517/mrr.2026.63)
Supplement: Supplementary file 1 [file mrr-5-3-23-SupplementaryMaterials.pdf]

Supplementary Materials

The microbiome bone axis in osteomyelitis integrating evidence from bone oral and gut microbiota

Chanon Kunasol[1,2], Chaloemwut Piyaphattachai[3], Jirawat Saengsin[3], Nipon Chattipakorn[1,2,4,5], Siriporn C. Chattipakorn[1,2,6]

[1]Neurophysiology Unit, Cardiac Electrophysiology Research and Training Center, Faculty of Medicine, Chiang Mai University, Chiang Mai 50200, Thailand.
[2]Center of Excellence in Cardiac Electrophysiology Research, Chiang Mai University, Chiang Mai 50200, Thailand.
[3]Pediatric Unit, Department of Orthopaedics, Faculty of Medicine, Chiang Mai University, Chiang Mai 50200, Thailand.
[4]Cardiac Electrophysiology Unit, Department of Physiology, Faculty of Medicine, Chiang Mai University, Chiang Mai 50200, Thailand.
[5]The Academy of Science, The Royal Society of Thailand, Bangkok, Thailand.
[6]Department of Oral Biology and Diagnostic Sciences, Faculty of Dentistry, Chiang Mai University, Chiang Mai, 50200 Thailand.

Correspondence to: Prof. Siriporn C. Chattipakorn, Neurophysiology Unit, Cardiac Electrophysiology Research and Training Center, Faculty of Medicine, Chiang Mai University Chiang Mai 50200, Thailand; Department of Oral Biology and Diagnostic Sciences, Faculty of Dentistry Chiang Mai University, Chiang Mai 50200, Thailand. E-mail: siriporn.c@cmu.ac.th

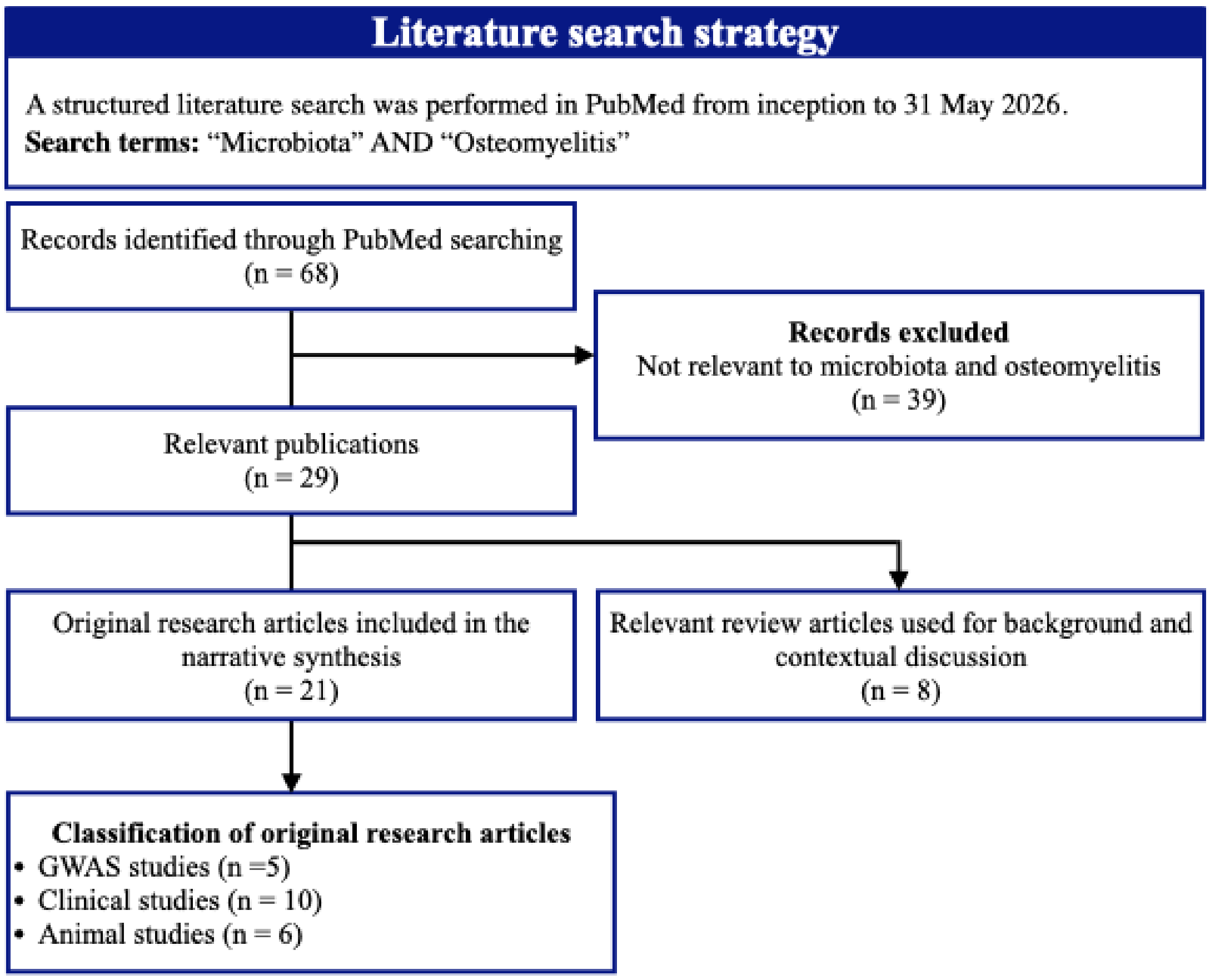

Supplementary Figure 1. Literature search and study selection process for this narrative review. The figure was created by the authors using Keynote.

Supplementary Table 1. Effect estimates from GWAS and Mendelian randomization studies investigating associations between microbiota and osteomyelitis

Taxa	Outcome	Direction	OR (95% CI)	P-value	Ref
**Skin Microbiome**					
Staphylococcus	Osteomyelitis	↑ Risk	1.067 (1.007–1.131)	0.027	(1)
Propionibacterium	Osteomyelitis	↑ Risk	1.068 (1.010–1.129)	0.021	(1)
Corynebacterium	Osteomyelitis	↑ Risk	1.072 (1.013–1.135)	0.016	(1)

Micrococcaceae	Osteomyelitis	↑ Risk	1.090 (1.027–1.157)	0.0045	(1)
Clostridiales	Osteomyelitis	↓ Risk	0.958 (0.926–0.991)	0.013	(1)
C. glutamicum	Osteomyelitis	↓ Risk	0.959 (0.933–0.987)	0.0037	(1)
Finegoldia	Osteomyelitis	↓ Risk	0.964 (0.931–0.998)	0.038	(1)
Betaproteobacteria	Osteomyelitis	↓ Risk	0.941 (0.902–0.980)	0.0038	(1)
**Gut Microbiome**					
Bacilli	Osteomyelitis	↑ Risk	1.361 (1.127–1.644)	0.011*	(2)
Bacteroidia	Osteomyelitis	↑ Risk	1.295 (1.051–1.594)	0.023*	(2)
Butyricimonas	Osteomyelitis	↑ Risk	1.227 (1.028–1.465)	0.023*	(2)
Lactobacillales	Osteomyelitis	↑ Risk	1.295 (1.051–1.594)	0.023*	(2)
Bacteroidales	Osteomyelitis	↑ Risk	1.295 (1.051–1.594)	0.023*	(2)
Lachnospira	Osteomyelitis	↓ Risk	0.666 (0.478–0.928)	0.023*	(2)
Bacteroidales S24.7	Osteomyelitis	↓ Risk	0.795 (0.656–0.965)	0.023*	(2)
Bacilli	Osteomyelitis	↑ Risk	1.36 (1.13–1.64)	0.022*	(3)
Butyricimonas	Osteomyelitis	↑ Risk	1.23 (1.03–1.46)	0.023	(4)
Coprococcus 3	Osteomyelitis	↑ Risk	1.37 (1.03–1.82)	0.029	(4)
Tyzzerella 3	Osteomyelitis	↑ Risk	1.15 (1.01–1.30)	0.036	(4)
Lachnospira	Osteomyelitis	↓ Risk	0.77 (0.61–0.98)	0.032	(4)

* p-values for Ref. 18 and 19 represent FDR-adjusted P-values (pFDR)

REFERENCES

1. Wang Y, Li Z. Mendelian randomization analysis unveils causal relationships between skin microbiota and osteomyelitis. Arch Dermatol Res. 2025;317(1):797.
2. Zhou Y, Yang Y, Zhu W, Kourkoumelis N, Wang Y, Chen Y, et al. Microbial Influences on Calcium-Phosphorus Homeostasis and Metabolic Bone Diseases: A Bidirectional Mendelian Randomisation Study on the Gut-Bone Axis. J Cell Mol Med. 2025;29(7):e70491.
3. Wang S, Yin F, Sun W, Li R, Guo Z, Wang Y, et al. The causal relationship between gut microbiota and nine infectious diseases: a two-sample Mendelian randomization analysis. Front Immunol. 2024;15:1304973.
4. Zeng W, Wu Y, Liang X, Cun D, Ma L, Zhang J, et al. Causal associations between human gut microbiota and osteomyelitis: a Mendelian randomization study. Front Cell Infect Microbiol. 2024;14:1338989.
